# First case of endometrial cancer after yolk sac tumor in a patient with Li-Fraumeni syndrome

**DOI:** 10.1186/s12905-023-02426-9

**Published:** 2023-06-21

**Authors:** Qiu-Lin Ye, Yue Qi, Juan-Juan Liu, Yue-Xin Hu, Yuan Lv, Bei Lin

**Affiliations:** Department of Obstetrics and Gynecology, Shengjing Hospital of China Medical University, No.36 Sanhao Street, Heping District, Shenyang, 110004 Liaoning China

**Keywords:** Li-Fraumeni syndrome, Yolk sac tumor, Endometrial cancer, *TP53*

## Abstract

**Background:**

Li-Fraumeni syndrome (LFS) is a rare autosomal dominant disease with high penetrance caused by a germline variant of *TP53* gene. We report the first case of endometrial cancer after yolk sac tumor with LFS.

**Case presentation:**

The presented female patient underwent right adnexectomy at age 23 because of a yolk sac tumor of the ovary. At the age of 27, the patient was diagnosed with endometrial adenocarcinoma, received cytoreductive surgery and chemotherapy. Given that her personal cancer history along with a strong family history of cancer, her father passing away from lung cancer at age 48 and her grandmother dying of ovarian cancer at age 50, the patient was referred for genetic counseling and testing. Genetic screening revealed a heterozygous pathogenic *TP53* c.844C > T, p.( R282 W) with NM_000546.5 variant, a class 5 (C5) variant. This is the first reported case of a yolk sac tumor accompanied by subsequent endometrial cancer that is associated with LFS.

**Conclusions:**

We reported a first case of an endometrial cancer after yolk sac tumor patient with a tumor family history of harboring the germline *TP53* pathogenic variation which expanded types of tumor that can be presented in patients with LFS. This case highlights the importance of genetic testing for patients with malignant tumors, as well as patients with a family history of malignant tumors. And our case highlights the necessity of screening for gynecologic tumor in LFS patients.

**Supplementary Information:**

The online version contains supplementary material available at 10.1186/s12905-023-02426-9.

## Background

Li-Fraumeni syndrome (LFS) tends to be a widespread, early-onset cancer associated with a germline variant in the *TP53* gene, located on the 17p13.1 chromosome that codes for p53, the most commonly inactivated protein in human cancer. Germline *TP53* variants most often occur in the DNA-binding domain, resulting in the production of a dysfunctional p53 protein [1]. The *TP53* gene encodes a transcription factor that controls the expression of multiple genes, which activate cell cycle arrest for genomic repair or apoptosis, depending on the level of DNA damage [2]. LFS is an autosomal dominant inheritance trait, and up to 25% of LFS is caused by de novo variants [3]. Tumor occurs in multiple organ systems is the clinical feature of LFS, which appears in a young age [4, 5]. Women with this syndrome have an almost 100% risk of cancer, while men have a 73% risk of cancer [3], the former has a higher penetrance rate mainly due to breast cancer [6]. However, the association of LFS with ovarian cancer and endometrial cancer (EC) remains underexamined. The purpose of this paper is to report a case in which a patient was first diagnosed with a yolk sac tumor (YST) of the ovary, then EC, and was later found to have LFS, which might be helpful in highlighting the importance of screening and surveillance in hereditary cancer-susceptibility syndromes.

## Case presentation

**Fig. 1:**
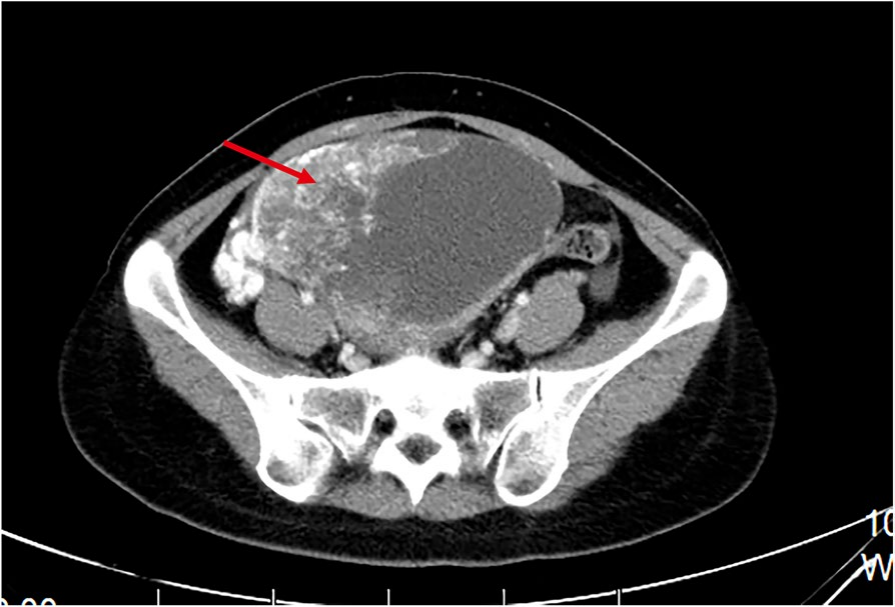
Computed tomography (CT) revealed a 14 cm solid cystic mass in the right adnexa

**Fig. 2:**
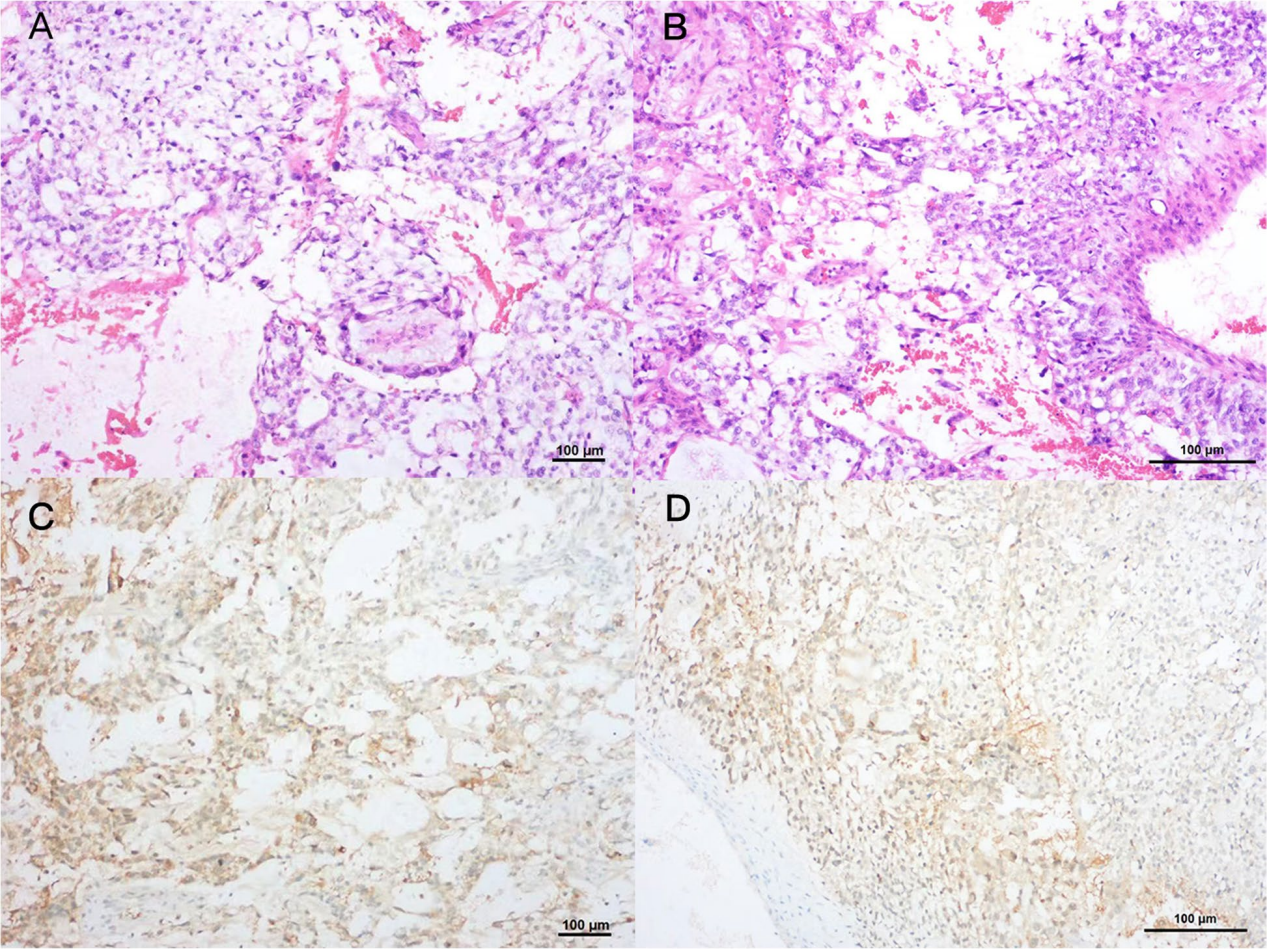
Yolk sac tumor pathology. Hematoxylin and eosin-stained photomicrographs, with original magnification 100 × (**A**) and 200 × (**B**), showing that myxoid stromal tumor cells are arranged in slices and microcapsules, with some eosinophilic globules visible. Immunohistochemistry, with original magnification 100 × (**C**) and 200 × (**D**), showing that tumor cells with 100% staining for AFP

- A 28-year-old female presented with right abdominal discomfort for two weeks in 2017 (age 23) without previous gynecologic abnormalities. Computed tomography (CT) revealed a 14 cm solid cystic mass in the right adnexa without other imaging abnormalities (Fig. 1). The level of serum alpha-fetoprotein (AFP) had risen to 1210 ng/mL, significantly higher than normal (0–9 ng/mL), while other tumor markers were within the normal range. She underwent right adnexectomy in ShengJing Hospital and was finally diagnosed with a yolk sac tumor (YST) of the ovary (Fig. 2). Ovarian germ cell tumors derive from ovarian germ cells and account for approximately 20% of all ovarian neoplasms. Only 1–2% of ovarian germ cell tumors are called malignant ovarian germ cell tumors. Yolk sac tumor represents the second most common tumor in the category of malignant ovarian germ cell tumors (25%) [7]. The immunohistochemistry (IHC) showed following results: AFP( +), CD30( ±), CK( +), oct4(-), SALL4( +). Intraoperatively, an encapsulated 15.0*14.0 cm cyst was observed in the right ovary, containing viscous fluid and jelly-like substance, which was adhered to the intestinal duct. No abnormalities were found in uterus, left ovary, bilateral fallopian tubes, appendix and intestinal tube. Intraoperative frozen pathology of the right ovarian mass was reported as malignant, considering yolk sac tumor. According to the patient's age and frozen pathological results, comprehensive staging laparotomy with fertility preservation function including cytological washings, omentectomy or omental biopsy, random peritoneal biopsies, and lymph node evaluation (pelvic and/or para-aortic lymphadenectomy or sampling) should be chosen. After informing the family of the frozen pathology results intraoperatively and consulting the family opinions about further surgery, they eventually refused to perform further comprehensive staging laparotomy, the operation ended up with right adnexectomy. After washing the pelvic and abdominal cavity thoroughly, the tumor without gross implantation in the pelvic cavity, omentum and appendix, the operation was not extended further. We performed CT scans of chest and abdomen a month after surgery, no metastatic images were found. She refused to receive the comprehensive staging laparotomy with fertility preservation function and received four cycles of postoperative chemotherapy with bleomycin, etoposide, and cisplatin (BEP) after the surgery. AFP decreased gradually after surgery and had already been in the normal range before the first chemotherapy. During the follow-up, uterine adnexal ultrasound was performed every 3 months, and abdominal CT was reviewed every 6 months. In addition to imaging examination, tumor markers were also tested and gynecological examination was performed, all of the results revealed no evidence of recurrence.

**Fig. 3:**
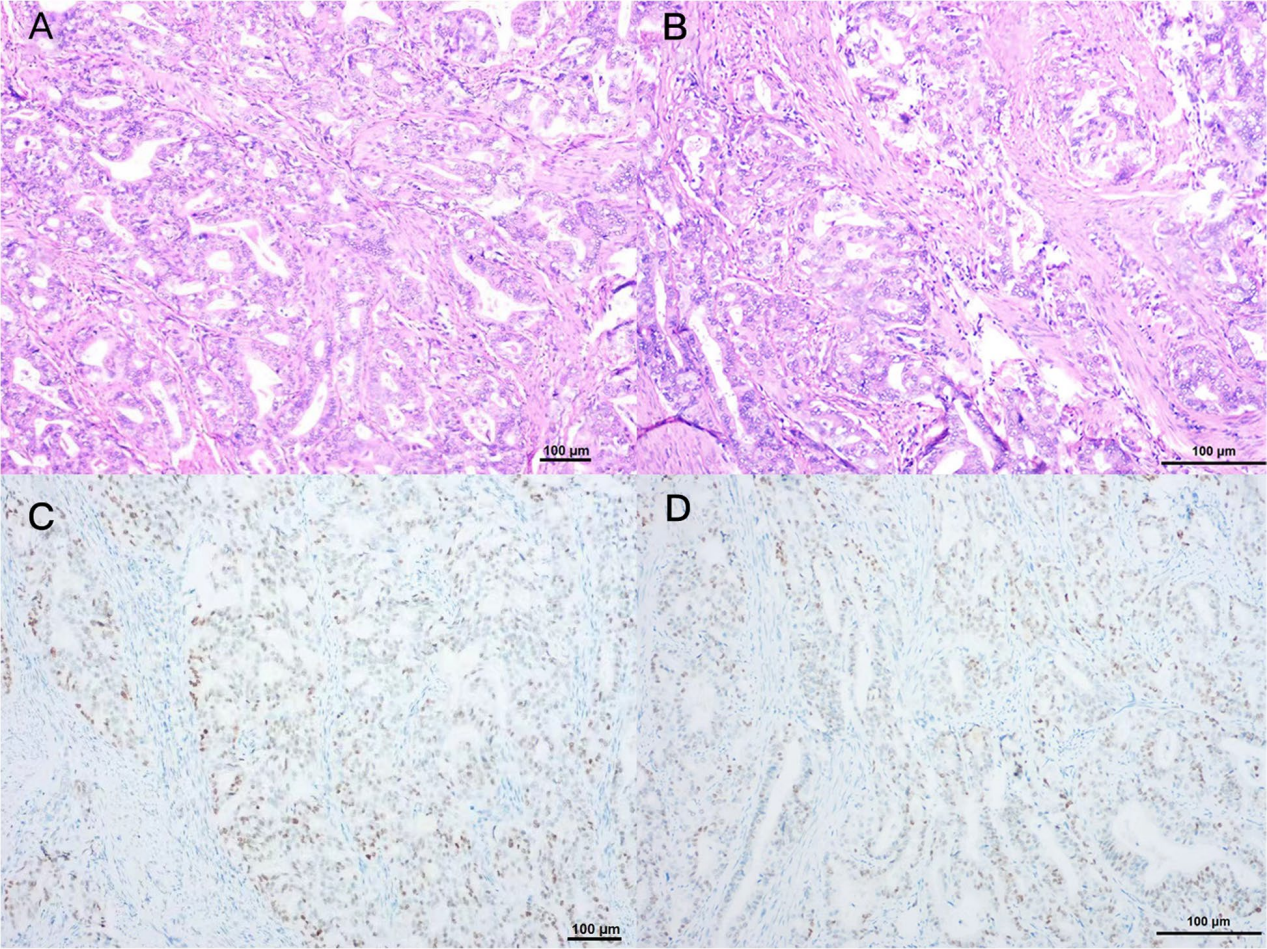
Endometrial cancer pathology. Hematoxylin and eosin-stained photomicrographs, with original magnification 100 × (**A**) and 200 × (**B**), showing that the cancer tissue is arranged in infiltrating, irregular adenoids. Immunohistochemistry, with original magnification 100 × (**C**) and 200 × (**D**), showing that tumor cells with 90% staining for p53

**Fig. 4:**
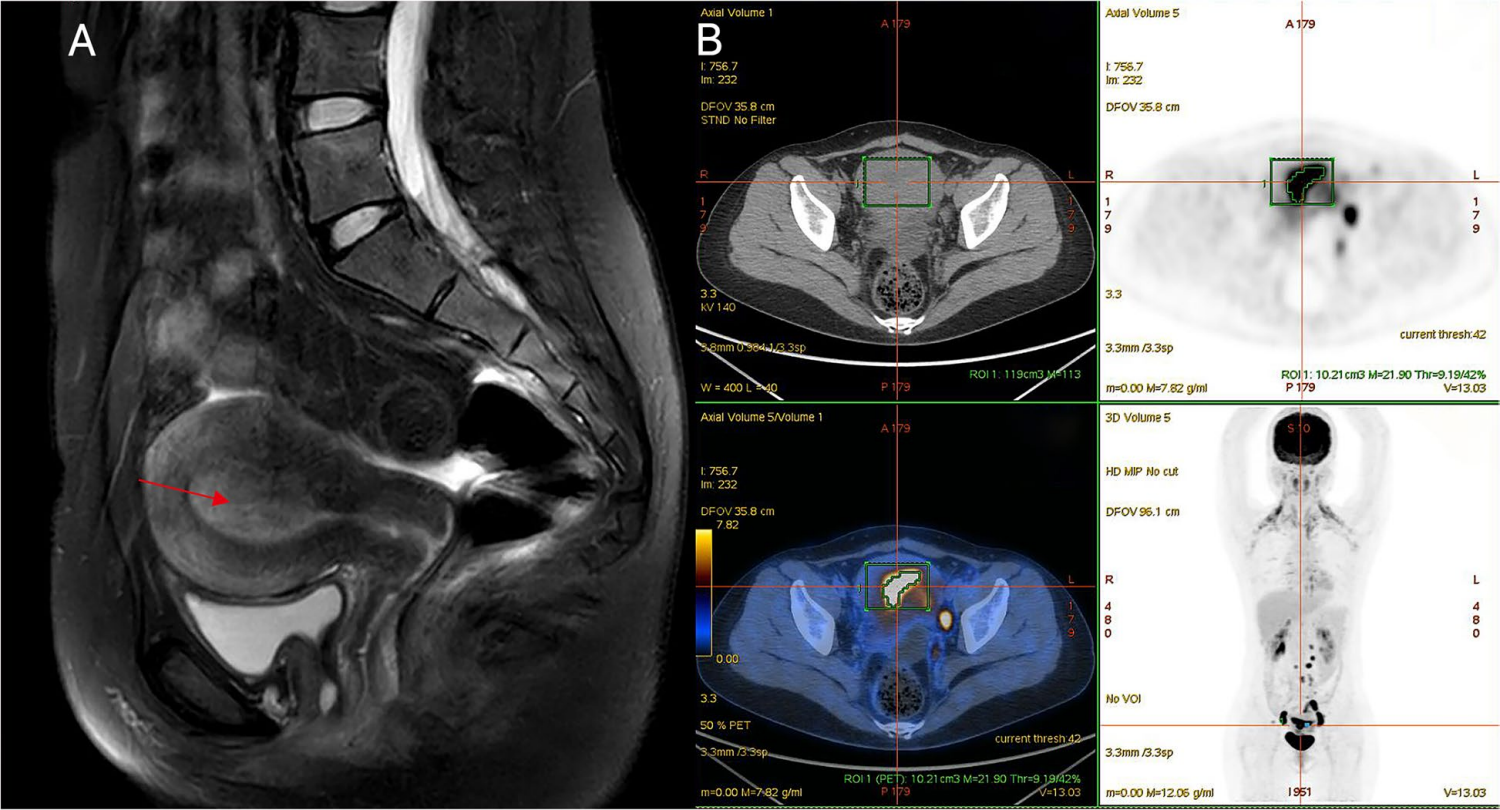
Imaging results of endometrial cancer. Magnetic resonance imaging shows that the endometrium is thickened, with a thickness of about 1.6 cm (red arrow), and the signal and enhancement are not uniform (**A**). Positron emission tomography/computed tomography shows increased fluorodeoxyglucose metabolism (SUVmax = 6.89) in the uterine cavity, consistent with endometrial cancer changes (**B**)

However, the patient began experiencing a prolonged menstrual period that lasted for 2 months in November 2020, while her periods had been normal before. She presented to Shengjing Hospital and was diagnosed with endometrial adenocarcinoma according to the results of hysteroscopy and curettage. Pelvic examination revealed a slightly enlarged uterus. Pathology reports showed a moderately differentiated endometrial adenocarcinoma (Fig. 3), the results of IHC were protein 53 (p53) (90% +), Ki-67 (30% +), cytokeratin 8/18 (10% +), estrogen receptor (ER) (80% +), progesteron receptor (PR) (95% +), MLH1( +), PMS2( +), MSH2( +) and MSH6( +) (Table 1). Magnetic resonance imaging (MRI) showed thickening of the endometrium (Fig. 4A), and subsequent positron emission tomography/CT (PET/CT) showed fluorodeoxyglucose uptake in the uterus, as well as in retroperitoneal lymph nodes, multiple lymph nodes on both sides of the pelvic walls, and pelvic peritoneum (Fig. 4B). The levels of tumor markers CA125 and HE4 were 123.8 U/mL, 165.0 pmol/L respectively.

**Table 1:** Comparison of immunohistochemistry results between preoperative curettage pathology and postoperative pathology of endometrial carcinoma

	Curettage Pathology	Postoperative pathology
ER	80% +	30% +
PR	95% +	40% +
Ki-67	30% +	30% +
P53	90% +	60% +
CK8/18	10% +	90% +
PAX2	negative	negative

*ER* Estrogen receptor, *PR* Progesteron receptor, + positivity, *CK* cytokeratin

**Fig. 5:**
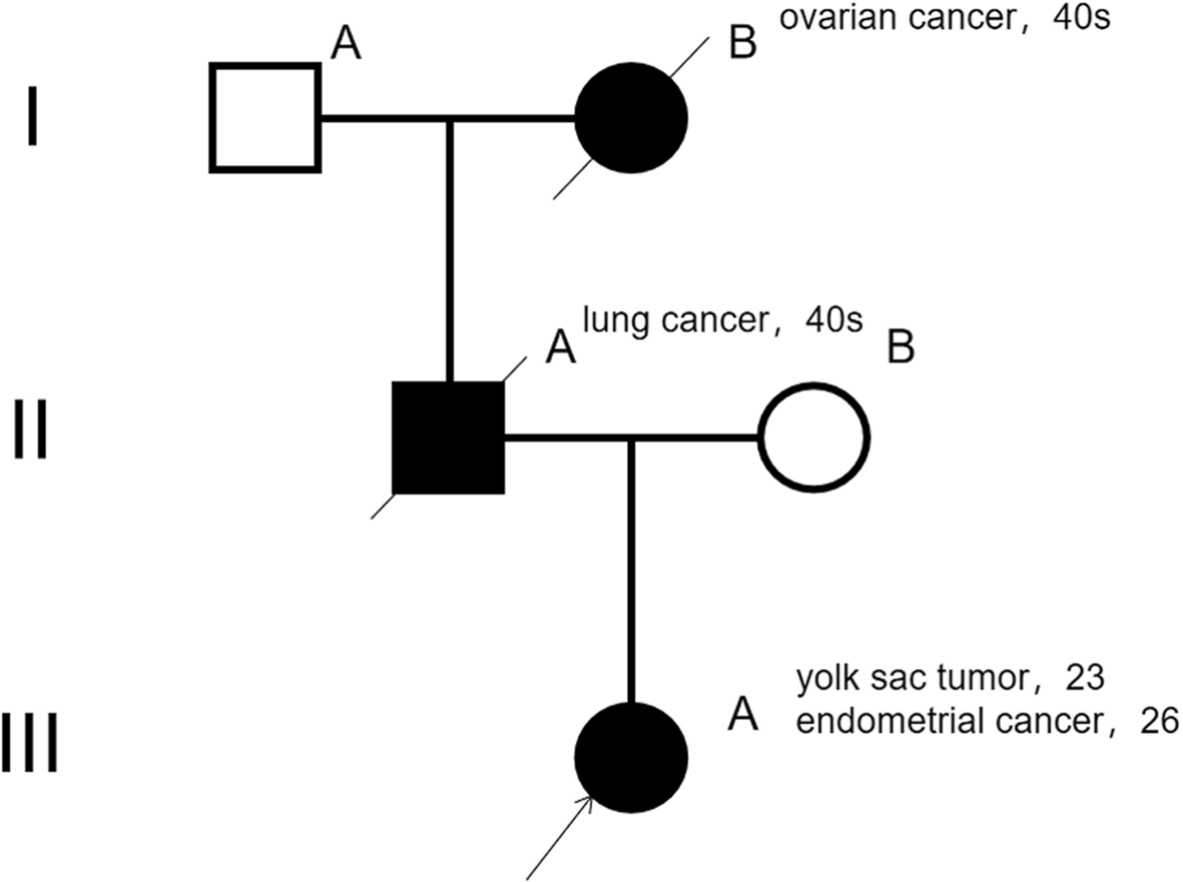
The pedigree of this family.The reported case (III A), her father (II A), and grandmother (I B). The cancer type and age of onset are listed beneath each affected family member

The patient underwent open cytoreductive surgery a month later, the final surgical procedures were radical hysterectomy, left adnexectomy, pelvic lymph node dissection, para-aortic lymph node dissection, pelvic and abdominal foci resection, omentectomy and appendectomy, tumor was completely excised without gross residual tumor. The pathological examination revealed moderately differentiated endometrioid adenocarcinoma in the deep muscle layer with cervical extension, omentum, peritoneum, intestinal surface lesion, left pelvic lymph node, presacral lymph nodes, and para-aortic lymph nodes metastases. The results of IHC were ER (30% +), PR (40% +), P53 (60% +), Ki-67 (30% +), cytokeratin 8/18 (90% +), MLH1( +), PMS2( +), MSH2( +), MSH6( +) and NapsinA negative (Table 1). According to the International Federation of Gynecology and Obstetrics (FIGO) guidelines, the patient’s surgical pathological staging was IVb. Her family history was investigated further: the patient stated that her father passed away from lung cancer at age 48, and her grandmother died of ovarian cancer at the age of 50 (Fig. 5).

She then received chemotherapy with bevacizumab, paclitaxel for injection (albumin bound) and carboplatin for 2 cycles. At the beginning of the third cycle of paclitaxel, the patient showed allergic symptoms including facial flushing, urticaria and labored breathing, so we changed the protocol to bevacizumab, liposomal doxorubicin, and carboplatin for 6 cycles. She eventually received 8 cycles chemotherapy and received maintenance therapy with bevacizumab q 21 days for 16cycles as a meta-analysis showed there were significant differences in progression-free survival (PFS) between patients receiving chemotherapy combined with and without bevacizumab [8]. There is little research evidence on the use of bevacizumab in EC while a clinical trial suggested that IHC for p53 alone or when integrated with sequencing for *TP53* mutations in EC for which bevacizumab is particularly beneficial in improving outcomes when combined with chemotherapy [9]. In light of the patient’s history of two gynecological malignancies and her family history of cancer, we conducted nextgeneration sequencing (NGS) of formalin-fixed paraffin-embedded (FFPE) EC tissue. This screening detected 688 genes related to the occurrence, development, treatment, and prognosis of solid tumors, and the test results were analyzed in detail, the complete list of genes is available as Supplementary Table 1 and can be obtained from the authors.

For molecular diagnosis, the Qiagen DNeasy Blood & Tissue Kit (Qiagen, Hilden, Germany) was used to extract genomic DNA (gDNA) from FFPE tissues, following the manufacturer’s protocol. Qubit (Life Technologies, Gaithersburg, Maryland, USA) and agarose gel electrophoresis were used to detect DNA concentration and quality. gDNA (250 ng) was used to construct the sequence library using the method described in previous literature [10]. The hybridization product was then purified, amplified, and quantified. Finally, 688 key cancer-related genes were identified with paired-end 100- and 8-bp barcodes on a MGISEQ-2000 sequencer following the manufacturer’s protocol. 1 germline variant and 6 somatic variants were detected, only the *TP53* c.844C > T, p.( R282 W) with NM_000546.5 was confirmed as a germline variation, the rest were verified as the somatic variations, Table 2 summarizes the somatic variant results.

**Table 2:** Summary of somatic variants in this patient. 6 somatic variants were detected in this case

Gene	Type of gene mutation	Gene subregion	Reference sequence	Amino acid change	cDNA change	Variant classification	Variant Allele Frequency(%)
*PTEN*	Somatic mutation	EX8	NM_000314.4	p.T319*	c.955_958delACTT	Class II	14.93
*CSDE1*	Somatic mutation	EX11	NM_001130523.2	p.R386Lfs*21	c.1157delG	Class II	9.09
*FGF12*	Somatic mutation	EX4	NM_021032.4	p.E168	c.502G > T	Class II	6.52
*ZFHX4*	Somatic mutation	EX2	NM_024721.4	p.L644I	c.1930C > A	Class III	5.17
*GID4*	Somatic mutation	EX1	NM_024052.4	p.A68del	c.203_205delCGG	Class III	1.12
*MAP3K4*	Somatic mutation	EX17	NM_005922.2	p.A1197V	c.3590C > T	Class III	0.62

**Fig. 6:**
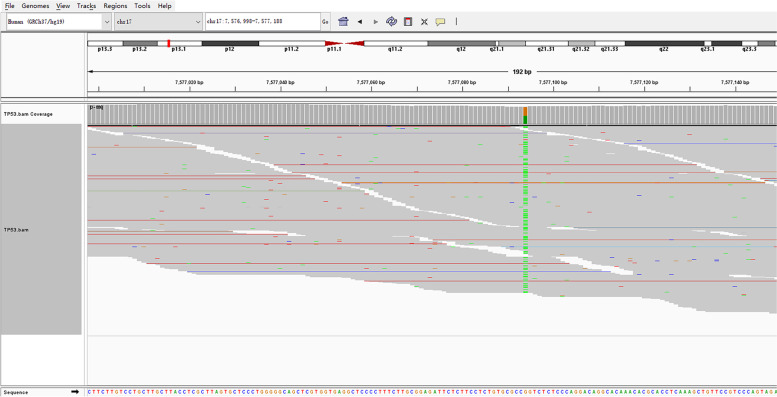
The integrative genomics viewer snapshot of *TP53* c.844C > T

*TP53* c.844C>T, p.( R282 W) with NM_000546.5 was found in a germline variant, a class 5 (C5) variant, inheritance of which is autosomal dominant. Her father and grandmother suffered from malignant tumor at a young age, it is very likely that her patrilineal family carried the *TP53* gene mutation and passed it on to the patient, she got YST accompanied by subsequent EC that is associated with LFS, these two tumors rarely occur in a patient with LFS, this case represented a new finding that extends the clinical scope of LFS; since her father and grandmother had passed away, family verification could not be performed, we confirmed the mutation as a germline heterozygous variation (Fig. 6).

The timeline of major events in the patient’s treatment is shown in Table 3. Variants in Polymerase Epsilon (*POLE*) gene were not detected, as the same as microsatellite instability. Through this germline analysis, we suggested her mother and other patrilineal relatives received the germline testing, which would help the whole family discover or eliminate the risk of tumor in time. Sanger sequencing was performed on her mother in order to rule out carrying the *TP53* variant during the follow-up, and the result was negative. It was further proved that her paternal family carried the *TP53* variant. She is undergoing gynecological pelvic examinations, tumor markers and regular imaging for the progression of her disease. In addition, regular gastroenteroscopy, head and abdominal CT scans are also included during follow up to rule out other tumors.

**Table 3:** Important dates in the case

Date	Event
10/2018	Began to develop symptoms of abdominal discomfort
11/2018	CT showed a solid cystic mass in the right adnexa
12/2018	Received the surgery of right adnexectomy and diagnosed with yolk sac tumor of the ovary
11/2020	Increased and extended period of menstruation for 2 months
01/2021	The result of the hysteroscopic curettage showed moderately-differentiated adenocarcinoma
07/2021	Genetic testing detected a germline *TP53* mutation

## Discussion and conclusions

Patients with LFS have a significant lifetime cancer risk [11–13]. Here, we report the first case of a YST accompanied by subsequent EC that is associated with LFS. In this case, genetic screening revealed a heterozygous pathogenic *TP53* variant (p.R282 W). LFS patients are prone to ovarian and endometrial cancers [14] while these two tumors rarely occur in LFS patients at the same time. As such, this case represents a new finding that extends the clinical scope of LFS. The histopathology of the two tumors in this case was different, and there was no direct association between them. In comparison to other tumor syndromes, such as hereditary breast-ovarian cancer and hereditary nonpolyposis colon cancer syndromes, each tumor should be considered individually in people with *TP53* germline variants. *TP53* variants are often inherited, and family history is still the key criterion for considering LFS [15].

*TP53* is the most mutated gene in tumors with some hot spots. In LFS, most *TP53* variants are located in the highly-conserved regions of exons 5–8 of the DNA-binding domain, especially in exons 7 and 8 [16]. The specific *TP53* variant in this patient is that a cytosine-to-thymine transition leads to a missense arginine-to-tryptophan transition at amino acid 282 (p. R282W) within exon 8. The R282W germline variant has previously been observed in cohorts that tend to develop mixed adenoneuroendocrine carcinoma of the gallbladder and breast cancer [17, 18]. The R282W variant has been regarded as a hotspot variant at both germline and somatic levels associated with poorer prognosis as compared to other pathogenic missense variants [19], this variant is included in the dbSNP database (rs28934574). The R282W mutant suppresses the expression of Kruppel-like-factor 17 (KLF17) which inhibits the promoters of epithelial mesenchymal transition-related genes, and thereby induced epithelial mesenchymal transition (EMT) [20]. An in vitro study discovered that R282W mutant was associated with miR-155 expression which promote cellular transformation and invasion [21]. The R282W mutant is associated with an earlier onset of familial cancers and poorer outcomes of cancer patients.

Pathological consequences of p53 variant include loss of normal p53 function, dominant-negative variants that can alter wild-type p53 function, and even a rare form of translocation defects with cytoplasmic accumulation and nuclear exclusion, particularly in certain regulatory domain variants [22]. In particular, when tumors show p53 expression by immunostaining, genetic testing for LFS should be performed, particularly in young patients. A research observed a high degree of concordance between *TP53* variantal status and p53 protein expression by immunostaining [23]. p53 immunostaining should be performed on all malignant endometrial biopsies or curettings, and the immunophenotype of a carcinoma represented in a biopsy or curetting, tends to be concordant with the matched resection specimen [24]. A cohort study evaluating the need for germline testing in young patients with p53 expression in tumors is warranted.

In addition, the current patient carried six types of somatic cancer variants, especially *PTEN,* frequently detected in EC and often appears in 80% of Cowden syndrome, has the most somatic copy number variations [25, 26]. *PTEN* is part of the PI3K/AKT/mTOR pathway regulation [27]. Loss of *PTEN* function in EC, via inactivating variant, deletion, or loss of protein expression, is associated with elevated levels of phosphorylated AKT [28]. The Cancer Genome Atlas database shows that the variation frequency of *PTEN* in EC was 66.42%. Loss or alteration of *PTEN* occurs in 45% of EC and is more commonly found in endometrioid EC than in other histological subtypes. A study showed that the *TP53* variant frequency in serous endometrial carcinomas (> 90%) differentiated them from the endometrioid subtypes (11.4%) [29], another research suggested that the most common histologic subtype of *TP53*-mutated endometrial carcinomas is uterine serous carcinoma [23]. However, 50% endometrioid tumors with a non-silent *TP53* variant also have non-silent variants in *PTEN*, compared to only 2.6% serous tumors with non-silent *TP53* variants, although *TP53* variants are not restricted to serous tumors, the co-existing *PTEN* variants in the endometrioid cases suggest a distinct tumorigenic mechanism [23], which is exactly what had happened in this case.

Ovarian tumors reported in cases of LFS usually correspond to common epithelial tumors [30]. It had also been reported that epithelial and mesenchymal components of ovarian carcinosarcoma, ovarian neoplasms of the sex cord-stromal type were concordancly associated with *TP53* germline variants [31]. However, there are few reports on YST associated with *TP53* germline variants. This paper reports for the first time, a LFS patient with a rare ovarian germ cell tumor. The current understanding of YST at the molecular level is very limited, despite recent cancer genomic characterization efforts. The genomic landscape, evolutionary pattern, and chemoresistance-related mechanisms of this disease are largely unknown, due to lack of molecular evidence in this kind of tumor, further researches are needed with clinical trials and studies dedicated to better understanding the rare gynecological tumor molecular profile and pathogenesis.

In the National Cancer Institute’s LFS study, Phuong L. Mai reported the risk assessment of the first and subsequent cancers and the annual cumulative risk of the first and second cancer in *TP53* variant carriers. The results showed that approximately 49% of people carrying with *TP53* variants developed a subsequent cancer within 10 years of the first cancer. The average age-specific risk of developing a second cancer was comparable to the risk of developing a first cancer [32]. Hisada reported that patients with a germline *TP53* variant had an increased risk of developing a second cancer; 30 years after the first cancer was diagnosed, the cumulative probability of developing the second cancer was 57% [33]. In this case, the interval between the two malignant tumors was only 4 years. This implies that LFS patients require regular follow-ups and physical examinations, regardless of how long they have been out of treatment. Clinical management of the patient may include monitoring other organs for tumor development [34].

Genetic counselling and predictive testing should be offered to patients fulfilling the classic LFS criteria as well as to their relatives, with intensified cancer screening if LFS is confirmed. This patient was very cooperative with our recommendation for genetic testing, and she understood that it is very important to her prognosis. As increasingly comprehensive genetic testing is provided to individuals who do not display a confirmed syndrome phenotype or family history, a wider range of aberrant expressions associated with germline variants of cancer-susceptibility genes may be realized. When pathology results show aberrant p53 patterns, as in this case, germline testing should be performed, particularly in young patients.

This study represents the first reported case of a young, female patient with LFS who developed EC after a YST, highlighting the importance of screening and surveillance in hereditary cancer-susceptibility syndromes. This case highlights the importance of genetic testing for patients with malignant tumors, as well as patients with a family history of malignant tumors. Genetic screening not only provides enhanced cancer monitoring to improve the prognosis of patients, but also supports individualized and risk-based treatment decisions.

## Supplementary Information

**Additional file 1:** **Supplementary Table 1.** The genes list of the 688 genes detected in this case.

## Data Availability

The datasets used during the current study available from the corresponding author on reasonable request.

## Abbreviations

LFS: Li-Fraumeni syndrome
EC: Endometrial cancer
YST: Yolk sac tumor
CT: Computed tomography
AFP: Alpha fetoprotein
IHC: Immunohistochemistry
BEP: Bleomycin, etoposide, and cisplatin
ER: Estrogen receptor
PR: Progesteron receptor
MRI: Magnetic resonance imaging
PET/CT: Positron emission tomography/CT
MMRd: Mismatch repair deficiency
FIGO: International Federation of Gynecology and Obstetrics
PFS: Progression-free survival
NGS: Nextgeneration sequencing
FFPE: Formalin-fixed paraffin-embedded
gDNA: Genomic DNA
POLE: Polymerase Epsilon
KLF17: Kruppel-like-factor 17
EMT: Epithelial mesenchymal transition
