# Supplementary material for: First case of endometrial cancer after yolk sac tumor in a patient with Li-Fraumeni syndrome
Source: BMC Womens Health. 2023 Jun 21;23:329. doi: 10.1186/s12905-023-02426-9 (PMC10286347; doi:10.1186/s12905-023-02426-9)
Supplement: Supplementary file 1 — Additional file 1: Supplementary Table 1. The genes list of the 688 genes detected in this case. [file 12905_2023_2426_MOESM1_ESM.docx]

**Supplementary Table 1 The genes list of the 688 genes detected in this case**

| ABCB1 | CCND2 | DSCAM | FGF3 | HNF1A | MAP3K13 | NHEJ1 | POU5F1 | RYBP | TAP2 |
| --- | --- | --- | --- | --- | --- | --- | --- | --- | --- |
| ABCG2 | CCND3 | DUSP4 | FGF4 | HOXB13 | MAP3K14 | NKX2-1 | PPARG | RYR2 | TBL1XR1 |
| ABL1 | CCNE1 | DUT | FGF6 | HRAS | MAP4K3 | NKX3-1 | PPM1D | RYR3 | TBX3 |
| ABRAXAS1 | CD74 | DYNC2H1 | FGF10 | HSD3B1 | MAPK1 | NLRP1 | PPP2R1A | SCG5 | TCF3 |
| ACSL3 | CD79B | E2F3 | FGF12 | HSD17B4 | MAPK3 | NOTCH1 | PPP2R2A | SDC4 | TCF4 |
| ACVR1 | CD274 | EDC4 | FGF14 | HSP90AA1 | MAPKAP1 | NOTCH2 | PPP4R2 | SDHA | TCF7L2 |
| ACVR2A | CD276 | EGFR | FGF19 | HSPA4 | MAX | NOTCH3 | PPP6C | SDHAF2 | TEK |
| ACYP2 | CDC27 | EF1AX | FGFR1 | ICOSLG | MB2W2 | NOT CH4 | PRDM1 | SDHB | TERT |
| ADGRA2 | CDC42 | EIF4A2 | FGFR2 | !D3 | MC1R | NPM1 | PRDM14 | SDHC | TET1 |
| AFF4 | CDC73 | ELAC2 | FGFR3 | DH1 | MCL1 | NQO1 | PREX2 | SDHD | TET2 |
| AJUBA | CDH1 | ELF3 | FGFR4 | IDH2 | MDC1 | NR4A3 | PRKAR1A | SEMA3C | TFE3 |
| AKT1 | CDH9 | ELOC | FH | IFNGR1 | MDH2 | NRAS | PRKCI | SESN1 | TGFBR1 |
| AKT2 | CDK4 | EME1 | FLCN | IGF1 | MDM2 | NSD1 | PRKD1 | SESN2 | TGFBR2 |
| AKT3 | CDK6 | EME2 | FLU | IGF1R | MDM4 | NSD2 | PRKDC | SESN3 | TIPARP |
| ALK | CDK8 | EML4 | FLNA | IGF2 | MECOM | NSD3 | PRKN | SETD2 | I MEM 127 |
| AMER1 | CDK12 | EMSY | FLT1 | GF2R | MED 12 | NT5C2 | PRPF40B | SF3B1 | TMPRSS2 |
| APC | CD KN 1 A | EP300 | FLT3 | IKBKE | MEF2B | NTHL1 | PRSS1 | SGK1 | TNFAIP3 |
| APOB | CDKN1B | EPCAM | FLT4 | IKZF1 | MEN1 | NTRK1 | PTCH1 | SH2B3 | TNFRSF14 |
| AR | CDKN1C | EPHA2 | FOXA1 | IL7R | MERTK | NTRK2 | PTCH2 | SH2D1A | TNFSF11 |
| ARAF | CDKN2A | EPHA3 | FOXL2 | IL10 | MET | NTRK3 | PTEN | SHOC2 | TOP1 |
| ARID1A | CDKN2B | EPHA4 | FOXO1 | INHA | MGA | NUDT18 | PTGS | SHPRH | TOP3A |
| ARID1B | CDKN2C | EPHB1 | FOXP1 | INHBA | MGMT | NUF2 | PTP4A1 | SHQ1 | TOPBP 1 |
| ARID2 | CDRT4 | EPPK1 | FRAS1 | INPP4A | MTF | NUTM1 | PTPN11 | SIPA1 | TP53 |
| ASXL1 | CDX2 | ERBB2 | FUBP1 | NPP4B | MKNK1 | NYAP2 | PTPRD | SLC7A8 | TP53BP 1 |
| ATAD2 | CEBPA | ERBB3 | FYN | NSR | MLH1 | PAK1 | PTPRO | SLC28A3 | TP63 |
| ATF1 | CETN2 | ERBB4 | G6PC | IRF2 | MLH3 | PAK5 | PTPRS | SLC34A2 | TPM3 |
| ATM | CFTR | ERCC1 | GAB2 | IRF4 | MMS19 | PALB2 | PTPRT | SLC45A3 | TRAF2 |
| ATR | CHD1 | ERCC2 | GABRA6 | RS2 | MPL | PARP1 | QKl | SLCO1B1 | TRAF7 |
| ATRX | CHEK1 | ERCC3 | GALNT12 | JAK1 | MRE11 | PARP2 | RAB35 | SLX1A | TRRAP |
| A UR KA | CHEK2 | ERCC4 | GATA1 | JAK2 | MS4A1 | PARP3 | RAC1 | SLX4 | TSC1 |
| A UR KB | CIC | ERCC5 | GATA2 | JAK3 | MSH2 | PARP4 | RAC2 | SMAD2 | TSC2 |
| AXIN1 | CLK2 | ERCC6 | GATA3 | JMJD1C | MSH3 | PAX5 | RAD21 | SMAD3 | TSHR |
| AXIN2 | COL11A1 | ERF | GATA4 | JUN | MSH4 | PAX8 | RAD50 | SMAD4 | TUBB3 |
| AXL | COL22A1 | ERG | GATA6 | KDM5C | MSH5 | PBRM1 | RAD51 | SMARCA1 | TYMS |
| B2M | COP1 | ERRF11 | GEN1 | KDM6A | MSH6 | PBX1 | RAD51B | SMARCA4 | U2AF1 |
| BABAM2 | CREB1 | ESR1 | GGH | KDR | MSl1 | PCDH9 | RAD51C | SMARCB1 | UGT1A1 |
| BACH1 | CREBBP | ETV1 | GID4 | KEAP1 | MSl2 | PDCD1 | RAD51D | SMARCD1 | UMPS |
| BAP1 | CRKL | ETV4 | GLI1 | KlAA 1549 | MST1 | PDCD1LG2 | RAD52 | SMO | UNC5D |
| BARD1 | CSDE1 | ETV5 | GNA11 | KIF1B | MST1R | PDGFRA | RAD54B | SMYD3 | UPF1 |
| BCL2 | CSF1R | ETV6 | GNAQ | KIF5B | MTAP | PDGFRB | RAD54L | SNCAlP | USP6 |
| BCL2A1 | CSMD3 | EWSR1 | GNAS | KlT | MTDH | PDK1 | RAF1 | SOCS1 | VEGFA |
| BCL2L1 | CTCF | EXO1 | GPS2 | KLF6 | MTHFR | PGR | RARA | SOD2 | VHL |
| BCL6 | CTLA4 | EXOC2 | GRB7 | KLHL6 | MT OR | PHF6 | RASA1 | SOS1 | VTCN1 |
| BCOR | CTNNA1 | EXT1 | GREM1 | KLLN | MTRR | PHOX2B | RB1 | SOX2 | WEE1 |
| BCR | CTNNB1 | EXT2 | GRIN2A | KMT2A | MUC6 | PIK3CA | RBBP8 | SOX4 | WRN |
| BIRC2 | CTNND2 | EZH1 | GRM3 | KMT2B | MUC16 | PIK3CB | RBM10 | SOX9 | WT1 |
| BIRC3 | CUL3 | EZH2 | GSK3B | KMT2C | MUS81 | PIK3CG | RECQL | SOX10 | WWTR1 |
| BLM | CUL4A | EZR | GSTP1 | KMT2D | MUTYH | PIK3R1 | RECQL4 | SOX17 | XAP |
| BMPR1A | CUL4B | FAM135B | H1-2 | KMT5A | MYB | PIK3R2 | REEP5 | SPEN | XPA |
| BRAF | CXCR4 | FAN1 | H2AX | KNSTRN | MYC | PIK3R3 | REL | SPINK1 | XPC |
| BRCA1 | CYLD | FANCA | H2BC5 | KRAS | MYCL | Pl M1 | RET | SPOP | XPO1 |
| BRCA2 | CYP2C8 | FANCB | H3-3A | LAMA2 | MYCN | PLAG1 | RFC4 | SPOPL | XRCC1 |
| BRCC3 | CYP2D6 | FANCC | H3-3B | LATS1 | MYD88 | PLCG2 | RHEB | SPRED1 | XRCC2 |
| BRD4 | CYP11B1 | FANCD2 | H3-4 | LATS2 | MYOD1 | PLK1 | RHOA | SRC | XRCC3 |
| BRF1 | CYP17A1 | FANCE | H3C1 | LHCGR | MYSM1 | PLK2 | RlCTOR | SRSF2 | YAP1 |
| BRIP1 | CYP19A1 | FANCF | H3C2 | LlFR | NABP2 | PLXNA1 | Rl T1 | STAG1 | YES1 |
| BTK | DAXX | FANCG | H3C3 | LlG4 | NBN | PMA lP1 | RNF43 | STAG2 | YWHAZ |
| C8orf34 | DCUN1D1 | FANCI | H3C4 | LRP1B | NCOA2 | PMS1 | ROS1 | STAT3 | ZBTB16 |
| CARD11 | DDB2 | FANCL | H3C6 | LRRK1 | NCOA3 | PMS2 | RPS6KA3 | STAT5A | ZFHX3 |
| CARM1 | DDR1 | FANCM | H3C7 | LRRK2 | NCOA4 | PNPLA3 | RPS6KA4 | STAT5B | ZFHX4 |
| CASP8 | DDR2 | FAT1 | H3C8 | LTK | NCOR1 | PNRC1 | RPS6KB2 | STK11 | ZMYM3 |
| CASR | DICER1 | FAT2 | H3C10 | LYN | NCOR2 | POLD1 | RRAGC | STK19 | ZNF2 |
| CBL | DIS3 | FAT3 | H3C11 | LZTR1 | NEGR1 | POLE | RRAS | STK40 | ZNF217 |
| CBLB | DMC1 | FAT4 | H3C13 | MALT1 | NEIL2 | POLG | RRAS2 | SUFU | ZNF703 |
| CBR3 | DNMT3A | FBXW7 | H3C14 | MAP2K1 | NF1 | POLH | RSPO2 | SUZ12 | ZNF770 |
| CBX4 | DNTT | FCGR2B | HDAC1 | MAP2K2 | NF2 | POLM | RTEL1 | SYK | ZNRF3 |
| CCDC6 | DOCK2 | FCGR3A | HGF | MAP2K4 | NFE2L2 | POLN | RUFY4 | TAF1L | ZRSR2 |
| CCNA2 | DOTH | FGD4 | HLA-A | MAP3K1 | NFKB1 | POLQ | RUNX1 | TAF15 |  |
| CCND1 | DPYD | FGF2 | HLA-B | MAP3K4 | NFKBIA | POT1 | RXRA | TAP1 |  |
